# Food Bloggers on the Twitter Social Network: Yummy, Healthy, Homemade, and Vegan Food

**DOI:** 10.3390/foods11182798

**Published:** 2022-09-11

**Authors:** Ladislav Pilař, Lucie Pilařová, Martina Chalupová, Lucie Kvasničková Stanislavská, Jana Pitrová

**Affiliations:** Department of Management, Faculty of Economics and Management, Czech University of Life Sciences Prague, 165 21 Prague, Czech Republic

**Keywords:** food blogger, healthy food, home-made food, vegan food, Twitter, social network analysis

## Abstract

Many people now consider social networking to be an indispensable tool. There are now over 4.6 billion social media users, who leave a digital footprint through their online interactions. These big data provide enormous research potential for identifying the social and cultural aspects of the monitored topic. Moreover, the use of social media platforms has been found to have an impact on eating habits. The analysis of these social networks is thus essential to understand the factors that influence eating habits. To this aim, we identified the main topics associated with food bloggers on Twitter using the Social Media Analysis based on the Hashtag Research Framework of 686,450 Tweets captured from 171,243 unique users from 1 January 2017 to 30 May 2022. Based on the analysis of communication on Twitter, the most communicated hashtags in the food blogger sphere were as follows: #yummy, #healthy, #homemade, and #vegan. From the point of view of communities, three major clusters were identified, including (1) healthy lifestyle, (2) home-made food, and (3) fast food, and two minor clusters were identified, namely, (4) breakfast and brunch and (5) food traveling.

## 1. Introduction

Social media platforms represent impactful channels of communication in our strongly digitized lives, and have been increasingly used by food marketers to facilitate participatory interaction [1,2,3]. Using high levels of visual complexity, food marketing communication on social media has the potential to create an overall positive audience [4,5]. In the list of the 100 overall Instagram and Twitter hashtags for likes published in 2022 by All Hashtag, #food and #vegan were included [6,7].

Considering that blogging on social media platforms is based on the shared experience of users, blogs represent a type of knowledge approaching direct practice [8]. Blogs on social media can be characterized as multimedia “guides to life” [9]. They present a unique personal view of life, as well as huge amounts of searchable data that are global and relatively resource-efficient [10]. The blogosphere, as communicative interactions of various posts, comments, trackbacks and hyperlinks among blogs in a chosen genre, is highly dynamic [11]. Moreover, due to the blogosphere’s decentralized character, it is impossible to state the exact number of blogs [12]. Bloggers on Instagram or Twitter typically focus on a single area, such as food, travel, beauty, politics, technology, and health, and topics related to food and nutrition are becoming increasingly prominent [13,14,15].

Food represents a crucial underpinning of human society due to its socializing function [16,17]. Traditionally, communication about food occurred in a top-down process, whereby renowned experts (by training and profession) instructed the general public on how to cook [18]. Social media platforms have made it possible for everyone to share information about their food-related interests with internauts who share their passion (Diemer et al., 2014), which results in co-creation of their “digital foodscapes” [19]. According to previous research [20], the most common motivation for creating a food blog is the love of food and willingness to care for oneself and the public through food. In other research [21], 75% of food bloggers reported creating content for their personal satisfaction, followed by recognition from relatives (29%) and recognition from other bloggers (27%). Social media posts about food reflect a complex cultural shift, and transform passive media consumers into active co-creators of media production [22], as they go beyond displaying and enacting food-centered stories [23]. As noted by previous authors [24], these posts have an increasingly domestic orientation and the most-used word to depict them is “day-to-day” cooking.

Current research has focused on a variety of dietary specificities of food blogs and their impact on customer behavior, such as clean eating blogs [14,25], healthy eating blogs [26,27,28], and vegan diet blogs [29,30]. The major impact of these social media influencers lies within their ability to redefine what is considered to be current and updated [31]. It is essential to understand the dominant voices that sound across these digital foodscapes, what kinds of discursive resources they use, and how they inhabit and nurture their growth [32]. Twitter food blogs can be places to find recipes [33], information on various types of diets [34], advice and influence about child feeding behavior [35], and instructions for older people on how to stay well by publishing advice on nutrition [36], among others. To summarize, social media platforms may influence consumer behavior in numerous ways; therefore, it is critical for businesses involved in the food industry to study the potential of the data they provide. These data could be used at multiple stages of the business decision-making process to help understand which issues and trends are evolving [37,38], and to identify opportunities and threats to derive knowledgeable implications, particularly those involving marketing, such as product development, innovation, brand engagement, and competitive intelligence [39].

### 1.1. Food-Choice Methodology Related Consumer Research

To promote both human and planetary health, experts from several domains have over the years produced conceptual models that address issues influencing food choice. To better understand how various factors are involved in and interact with one another during the decision-making process, a multidisciplinary approach is necessary [40].

From a psychology perspective, most daily decisions are made without much thought or effort and are based on our experiences, feelings, and intuition [41]. However, most consumer science practices today require people to think consciously about their actions and behaviors, which could render the collected data less valid and reliable [42,43]. Methods based on direct questions can lead to biased, socially desirable, and over-rationalized responses, even if unintentional [44], and yet questionnaire surveys are still a frequently used tool in food choice research [45].

Each research method has its strengths and limitations. The greatest limitations of questionnaire surveys are the low response rate (sample size) and their time-consuming nature [46]. For example, previous studies implementing the Food Choice Questionnaire reported samples ranging from 121 to 5752 respondents (for example: *n* = 121 [47], *n* = 273 [48], *n* = 525 [49], and *n* = 5752 [50]). That said, questionnaire surveys allow personal data about respondents to be obtained, and thus to assess differences between individual segments in terms of socio-demographic characteristics.

In the context of social network analysis, questionnaire surveys have both advantages and disadvantages. Existing research in the food domain has reported samples of more than 100,000 social media platform users (for example, *n* = 427,936 [51], 313,883 [52], and 168,134 [53]). Yet, there is an absence of socio-demographic data. Based on previous research, the comparison of results from individual-based research approaches could be the optimal way to understand the individual determinants of food choice behavior.

### 1.2. Research Opportunities and Importance of Social Network Analysis

At present, there are over 4.6 billion social network users [54], and this is expected to increase to 5.85 billion social network users by 2027. If we compare these data with the predicted global population in 2027, approximately 70% of the population will use social networks (in 2022, this was 57%) [55].

Social network users are no longer just passive recipients of messages, but also creators of active and passive digital footprints through activities on social networks. An active digital footprint is primarily about creating content that communicates values, experiences, attitudes, and opinions [56,57,58,59]. This opens up the possibility of using this digital footprint for scientific and research purposes by analyzing communication on social networks. The importance and topicality of social network analysis has been demonstrated in research on a variety of topics, such as tobacco [60], sustainable tourism [61], organic wine [62], and smart home adoption [63].

Analysis of communication on social networks in the field of food is very important, mainly because these platforms affect consumers’ everyday lives in many ways, including dietary decisions and food preferences [51,64]. Understanding the factors that influence food choices is essential to the successful translation of dietary objectives into consumer behavior, business marketing, and health policy [53,65]. So-called “digitized food” has occupied all social media platforms, and plays a main role in facilitating the construction of contemporary digital communities and food-based marketing [32]. Positioned within the context of recent debates in the field of food marketing communication [66,67], the present study mapped part of the digital foodscape through identification of a distinct grouping of online food voices on Twitter. Our findings shed light on Twitter food communities and their shared values, including trending issues, which could be useful for food businesses in the development of their social media marketing activities.

### 1.3. Social Media Analysis Based on Hashtag Research (SMAHR) Framework

The uniqueness of the SMAHR framework stems primarily from the fact that this framework is focused on the social media analysis while employing social network analysis methods, such as Frequency, Eigenvector centrality, Community analysis and modularity [68]. With these specifics, the SMAHR is a competitive framework that may be utilized as an alternative framework in the field of social media analysis, most of which are primarily focused on semantic and sentiment analysis [69].

Hashtag analysis provides additional information on another method of social media analysis with a focus on Image or text analysis.

(a) Image analysis is a method, where we classify individual objects in the image using machine learning models. This method is more suitable for “image-oriented social media” such as Instagram [70].
(b) Text analysis is focused on the text part of message. Frameworks focusing on social media analysis applying natural language processing may fail to detect specific types of information since the report from which the hashtags are removed may be devoid of information. Furthermore, sarcasm is frequently utilized in the text, which the Natura Language algorithm finds difficult to recognize [71].

Based on the aforementioned aspects, the SMAHR framework provides a tool for research triangulation, which has already been proven in previous work [65,65,72,73].

### 1.4. Research Gap

Our findings illustrate that social media data analysis can provide highly useful insights into a breadth of related views, and also support those obtained using traditional methods.

### 1.5. Research Question and Aim of the Study

Based on the preceding literature, we asked the following research question: “What do food influencers say, when they tweet about food?”. The aim of the study was to identify the main topic associated with food bloggers on Twitter using the Social Media Analysis Based on Hashtag Research (SMAHR) Framework.

The paper is structured as follows: the Introduction of this paper offers a brief theoretical background about food marketing communication on social media, an overview of our research approach and research opportunities, and the importance of social media analysis. In the Materials and Methods, we describe the process of SMAHR. The Results and Discussion report the most communicated hashtags used by food bloggers on Twitter. Our community analysis presents further insights into the communication on social networks by identifying communities and their size, which also reveals the most interesting issues in Twitter food debates. We also identified the most communicated individual diet on the Twitter social media network. In the Conclusion, we clarify that food marketers could use Twitter effectively for their marketing activities, especially in connection with identified communities and trending topics.

## 2. Materials and Methods

The SMAHR Framework, initially developed for hashtag analysis, was used to analyze the data [68]. On Twitter, the hashtag refers to the part of the Tweet depicted by the hash “#” symbol. Hashtags have two basic functions on social media: first, as a filter to show messages according to a selected topic [74], and second, as a way to place values, experiences, attitudes, and opinions at the center of the message [56,57,58,59]. In the case of a food blogger, the hashtag #veganfood can be used to highlight the vegan food value of the message, which may not be apparent from the text and photography.

**Figure 1:**
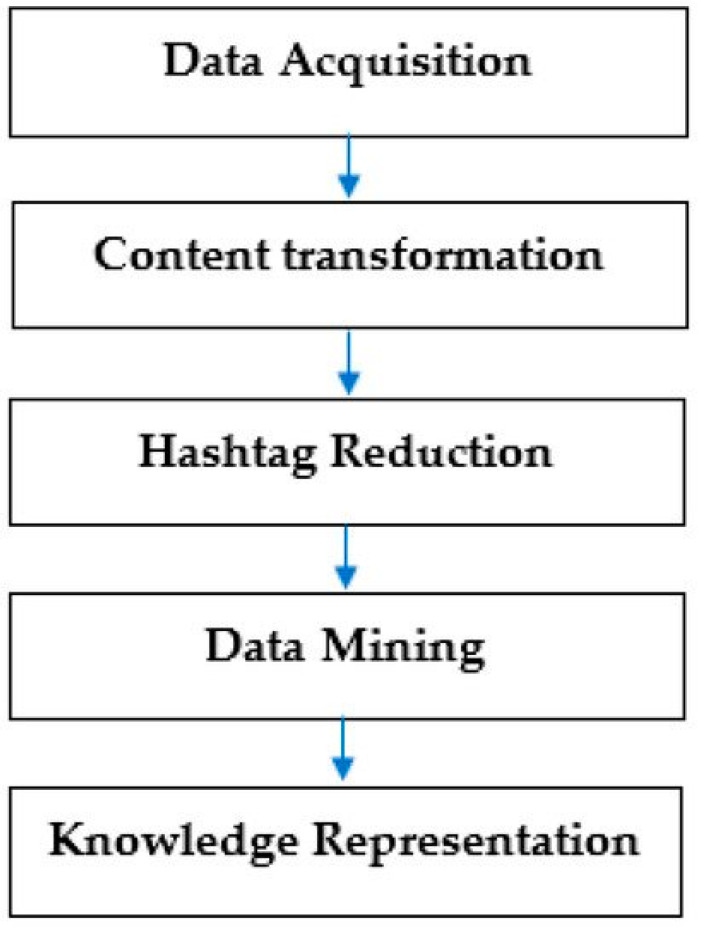
Process of Social Media Analysis using the SMAHR Framework.

**Figure 2:**
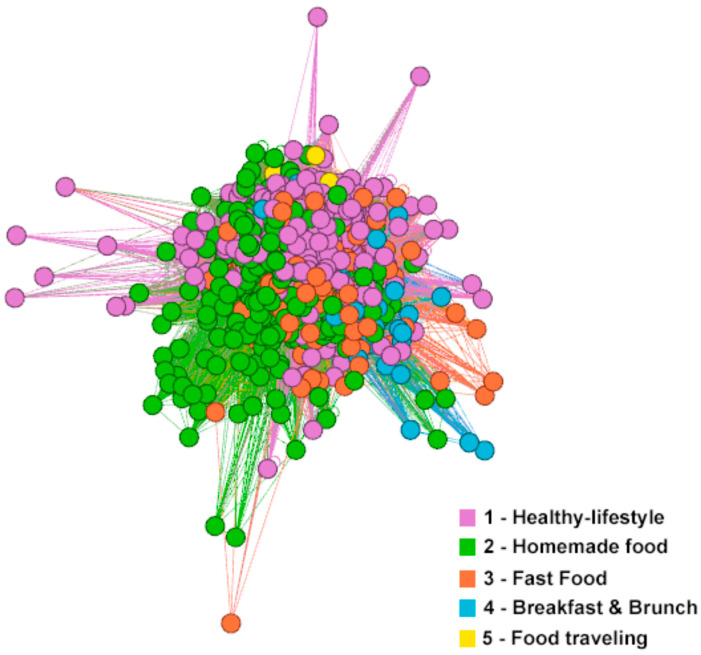
Community visualization in the area of food blogger on Twitter.

The SMAHR Framework has been successfully used in studies on farmers’ markets [65], organic foods [52], corporate social responsibility [73], sustainability [39], gamification [75,76], and healthy food [51,53]; the SMAHR Framework-based data analysis process consists of the five following steps (see Figure 1):

(1) Data acquisition: The Twitter API [77] was used to extract messages (Tweets) from the Twitter network. Tweets were collected in the time period between 30 May 2017 and 30 May 2022 (a 5-year period). In total, 686,450 Tweets with the hashtag #foodblogger were captured from 171,243 unique users by the Python script [78] during that period. This dataset contained all messages sent to the Twitter social network that included the hashtag #foodblogger during the monitored period.
(2) Content transformation: As our study was focused on hashtags, we excluded any phrases that did not start with the hashtag symbol (“#”). This resulted in a dataset consisting only of hashtags (i.e., words beginning with #). Subsequently, all uppercase characters were changed to lowercase letters to eliminate any duplications (for example, the program could interpret #Healthy, #healthy, and #HEALTHY to be three different hashtags). Then, a last change was made to separate strings of associated hashtags, such as “#healthy#organic,” which became “#healthy; #organic.” The data were imported into Gephi 0.9.3, and a hashtag corpus based on the interdependence of hashtags was developed (see Figure 2). Gephi is an open-source software for network visualization and relationship between nodes (hashtags) exploration [79].
(3) Hashtag reduction: Hashtag reduction was required in order to eliminate micro-communities prior to undertaking the community and modularity study. An abundance of hashtags, including local hashtags such as “#dallas” and “#dallasmicrocommunities,” creates much noise.
(4) Data mining: The hashtag network was described using the data mining methods listed below:
  (a) ***Frequency:*** A frequency is a number representing the frequency of hashtags in a network.
  (b) ***Eigenvector centrality:*** This metric reflects the impact of hashtags in a network and is an extension of degree centrality. Eigenvector centrality is calculated based on the premise that links to hashtags with high degree centrality values have a larger impact than links with similar or lower degree centrality values. A hashtag with a high eigenvector centrality value is connected to a large number of hashtags with a high degree centrality value. The eigenvector centrality was determined as follows:
    (1) $$x_{v}=\frac{1}{\lambda }\underset{t\in M\left(v\right)}{\sum }x_{t}=\frac{1}{\lambda }\underset{t\in G}{\sum }a_{v,t}x_{t}$$
    where *M*(*v*) denotes a set of adjacent nodes and *λ* is the largest eigenvalue. Eigenvector *x* can be expressed by Equation (2), as follows:
    (2) Ax=λx
  (c) ***Community analysis and modularity value:*** The most convoluted networks feature hashtags that are more closely related to one another than to the rest of the network. Communities are groups of such hashtags [80]. Modularity is an index that measures the cohesiveness of communities inside a network [81]. The goal of this analysis is to find hashtag groups that are more strongly linked than other hashtag communities. High modularity networks demonstrate significant relationships between hashtags within the community, but fewer links between hashtags in different communities [82]. Based on one modularity detection study [83], the community analysis then determines the number of various communities in the network, as follows (see Equation (3)):
    (3) $$\Delta Q=\left[\frac{\sum_{in}+2k_{i,in}}{2m}-{\left(\frac{\sum_{tot}+k_{i}}{2m}\right)}^{2}\right]-\left[\frac{\;\sum_{in}}{2m}-{\left(\frac{\sum_{tot}}{2m}\right)}^{2}\right]$$
    where ∑*_in_* is the total number of weighted links inside the community, ∑*_tot_* is the sum of weighted links incident to hashtags in the community, *k_i_* is the sum of weighted links incident to hashtag *i*, *k_i,in_* is the sum of weighted links going from *I* to hashtags in the community, while *m* represents the normalization factor as the sum of weighted links for the entire graph.
(5) Knowledge representation: The use of visualization tools to represent the outcomes of data mining is known as knowledge representation. Knowledge is represented through the synthesis of individual values and outputs from the data assessment process.

## 3. Results and Discussion

First, the frequency of individual hashtags in connection to Food Bloggers was analyzed (see Table 1). 

As shown in Table 1, the hashtags can be divided into two categories:

**(1)**  **Hashtags that are broad categorizations of a topic, such as #food or #blogger**

As shown in Table 1, many hashtags used in the area of “food bloggers” on Twitter are essentially synonyms. In second and third place were the hashtags #foodie and #food. These hashtags characterize the content of the message—food—as do the hashtags in fifth place (#foodphotography) and in twelfth place (#foodie). These hashtags are expected in the field of food, as is the hashtag #foodporn [84]. In recent years, #foodporn has become a trend in which social media users photograph their meals before or after consumption and upload them on the social networks [85]. The aim of these hashtags is to receive public recognition in the form of likes, comments, and shares [86,87]. The hashtag #foodstagram in ninth place identifies a profile that specializes in food and from which one can expect more food news. This has the same meaning as the tenth-ranked hashtag, #instafood, the fourteenth-ranked hashtag, #foodblog, and the twenty-sixth-ranked hashtag, #blogger.

**(2)**  **Hashtags identifying the characteristics of a given Tweet**

In sixth and seventh place were the hashtags #yummy and #delicious. These hashtags express the positive assessment of food in terms of taste [53]. In eleventh place was the hashtag #healthyfood, which describes the characteristics of food [51,53], as does the hashtag #healthy, which placed twenty-fourth and has the same meaning. This was followed by the hashtag #homemade in sixteenth place, which expresses the characteristic of home-made production, and, in nineteenth place, the hashtag #recipes, which indicates that the Tweet contains a recipe for the food presented in a post. In terms of the three basic meals of the day (breakfast, lunch, and dinner), food bloggers most often referred to dinner (see Table 2).

The hashtag #vegan placed twenty-fifth, and refers to a vegan diet. In general, a diet is a certain food selection chosen by an individual or group. This can either be a selection of foods that they want to eat, or that they do not want to eat. Dietary choices are frequently influenced by a range of variables, such as ethical and religious views, environmental perspectives, animal welfare, therapeutic needs, and weight control. The following three basic diets were found among the top 1000 hashtags: #vegan, #vegetarian, and #glutenfree (see Table 3).

Food choices have a direct impact on our physical and mental health through consumption, as well as an indirect impact on how we view ourselves and how others view us in terms of nutritional trends, our relationship with the environment, and animal welfare [88,89,90,91,92]. Influencer marketing is a very important part of shaping the image of the world, and young people in particular, who spend an average of 3.2 h/day on social networks [93], are greatly influenced by this communication.

It is possible to identify positive communication in the area of food using an analysis of communication on Twitter, because the most communicated characteristics are yummy, healthy, home-made, and vegan. 

Our results confirmed that the vegan market, which encompasses not only food but also cosmetics, apparel, and entertainment, is one of the largest consumption trends and is gradually increasing [94]. Veganism is an ever more popular lifestyle philosophy that aspires to abolish all types of animal exploitation and cruelty for food, clothing, and any other purpose [95].

When looking at meat, we identified the following types of meat according to their labeling in Tweets. Poultry was mentioned the most often (16,165 posts), followed by seafood (6333 posts) and beef (4657 posts). For more information, see Table 4.

### 3.1. Community Analysis

Community analysis provides a different method for analyzing communication on social networks. The following five communities were extrapolated from the community analysis: home-made food, healthy lifestyle, fast food, breakfast and brunch, and food traveling (Table 5).

The largest community was the “healthy lifestyle” community, which contained hashtags that were associated with areas such as healthy lifestyle, vegan, healthy eating, vegetarian, gluten free, organic, and diet. This community is focused on a healthy lifestyle that users associate with a vegan, vegetarian, and gluten-free diet, which has been supported by prior research into the perception of healthy and organic food [51,52,53].

The second largest community was “home-made food”. This community contained hashtags that were focused on healthy, home-made food and cooking. This community also included the hashtag #dinner, which indicates that home-made food was mostly served as dinner. Home-made food is food prepared at home and is associated with healthy characteristics [51].

The third community was “fast food”. This community included the hashtags #pizza, #pasta, #burger, #delivery, #yummy, #pizzatime, #cheatdate, and #delicious. In this fast food area, food bloggers presented food as “yummy”. This community comprised 18.94% of all communication, and was partially connected with the food traveling community (see Figure 2). The use of this community in the field of healthy food lifestyle can be explained by the fact that healthy food bloggers sometimes show that they eat unhealthy food; this allows them to show their human side, remind others that a diet is a personal journey, and that so-called “cheat days” are sometimes necessary [96]. This behavior can bring many positive reactions [96].

The fourth community was focused on “breakfast & brunch”, and contained the hashtags #cake, #sweet, #chocolate, #coffee, #baking, #cook, #desserts, #brunch, and #breakfast. This community was connected with the “fast food” community.

The last, fifth community was the “food traveling” community, which concerns the communication of food consumed while traveling by food bloggers. This community includes the hashtags #travel, #travelblogger, #travelgram, #foodtravel, #traveler, and #travelfoodblog.

The low polarity of individual clusters was identified based on a visual analysis, which was supported by the modularity value of 0.122. Individual communities were not polarized among themselves, as is the case, for example, with communication on political topics [97]. 

Practical implication

The practical implications can be divided into three following areas:

(1) Consumer behavior

Community analysis allowed us to detect clusters of potential customers, the most associated hashtags offer primary orientation in their buying choices. The largest identified community was “healthy lifestyle”, associated with the hashtags vegan, healthy eating, vegetarian, gluten free, organic, and diet. This presents a signal for food businesses with regard to the food purchases of customers willing to adopt a healthy diet.

(2) Business Marketing

The most tweeted meal of the day is dinner. Since the basic characteristic of food bloggers is home preparation, it can be assumed that the most frequently prepared meal at home is dinner (or that the greatest interest in home-prepared food is dinner). This can be used by marketing communication of the offered product as usable (suitable) for dinner, similarly to vegan diets (increase the offer of vegan products or for products that meet the characteristics but are not presented as such, and present them as vegan).

(3) Healthy Policy

The most consumed meat is poultry. Either a campaign can be implemented to draw attention to the fact that poultry meat is full of antibiotics (but that would probably require a deeper insight into the issue) or a campaign could be implemented to support the consumption of fish, since research has shown that they are not given the attention that is warranted from a health perspective.

### 3.2. Limitations

Social media analysis has strong research potential because of the expanding social media usage trends; however, several study limitations deserve attention. The first research limitation is related to the use of the SMARH framework [68], which only focuses on hashtags. 

The second research limitation is usage of only one social network—Twitter. Every social network has its audience. Unfortunately, as a result of the Cambridge Analytica data scandal in 2018, Meta stopped the API for Facebook and Instagram [98].

The third limitation is the lack of resolution of geolocation, in that we employed an analysis of global communication without information about locality.

The fourth limitation is the 5-year time series and the period of the COVID-19 pandemic. The COVID-19 pandemic has affected people’s behavior in many ways. One of which is certainly the food behavior of some users. Currently (2022), it is not possible to determine whether we are in a post-COVID-19 pandemic period, or whether we are still in the midst of the COVID-19 pandemic. Future studies based on this limitation are created in the following chapter.

### 3.3. Future Research

Following our analysis of global communication results, further research should aim to identify regional specifics that are associated with these global communication results. Another potential research direction is the analysis of communication on other social networks, such as Instagram, TikTok, and LinkedIn, in case of API opening for free download of data.

Following the COVID-19 pandemic, it would be appropriate to conduct research into behavioral changes related to the pandemic with the endowment of hindsight (3–5 years).

## 4. Conclusions

Our analysis of communication on the social network Twitter in the domain of food bloggers revealed that this area was mostly associated with the topics of “healthy food” and “healthy lifestyle”, followed by the topic of “home-made food”. The largest identified community was “healthy lifestyle”, associated with the hashtags vegan, healthy eating, vegetarian, gluten free, organic, and diet. This presents a signal for food businesses with regard to the food purchases of customers willing to adopt a healthy diet. Hashtags that were most communicated in connection with food bloggers were #yummy, #healthy, #homemade, and #vegan (synonyms are omitted here), which support research focused on healthy food in the area of increasing interest in homemade and vegan products, which is an important finding in the area of marketing communication of products to customers. Moreover, three major communities were identified (healthy-lifestyle, home-made food, and fast food), and two minor communities were identified (breakfast and brunch and food traveling). When focusing on the selection of individual diets, the vegan diet was the most communicated diet in connection with food bloggers, followed by the vegetarian diet and gluten-free diet. In terms of meat choice, poultry was the most popular. This finding again supports the growth of support for vegan products, which can be used both in strategic marketing in the area of communication and in strategic management in the area of product portfolio differentiation.

## Figures and Tables

**Table 1:** Hashtags tweeted in connection with the hashtag #foodblogger on Twitter.

No.	Hashtag	Frequency	Eigenvector Centrality	No.	Hashtag	Frequency	Eigenvector Centrality
1	#foodblogger	686,450	1	16	#homemade	69,514	0.981272
2	#foodie	319,361	0.999088	17	#tasty	68,151	0.985122
3	#food	291,155	0.999291	18	#foodgasm	67,723	0.984966
4	#foodporn	202,342	0.996423	19	#recipes	62,243	0.96859
5	#foodphotography	157,368	0.994621	20	#dinner	59,555	0.989529
6	#yummy	142,153	0.991842	21	#love	46,012	0.982301
7	#delicious	129,975	0.991128	22	#foodpics	44,557	0.983585
8	#foodstagram	128,112	0.991433	23	#instagood	43,199	0.983739
9	#foodlover	123,272	0.992486	24	#healthy	35,465	0.972448
10	#instafood	101,580	0.990388	25	#vegan	35,122	0.981412
11	#healthyfood	81,997	0.983406	26	#blogger	33,071	0.987989
12	#foodies	81,501	0.992421	27	#chef	32,989	0.981213
13	#recipe	79,525	0.976214	28	#lunch	32,267	0.985012
14	#foodblog	78,431	0.991668	29	#eat	32,153	0.974965
15	#cooking	72,079	0.986917	30	#foods	31,029	0.975571

**Table 2:** Meals of the day tweeted in connection with the hashtag #foodblogger on Twitter.

Meals of the Day	Frequency
Breakfast	24,231
Brunch	7327
Lunch	32,267
Dinner	59,555
Snack	8008

**Table 3:** Type of diet published in connection with the hashtag #foodblogger on Twitter.

Type of Diet	Frequency
Vegan diet	32,153
Vegetarian diet	12,363
Organic food diet	10,117
Gluten-free diet	7483
Weight loss diet	3261
Clean eating diet	2907
Low-carb diet	1235
Dairy-free diet	1757
Sugar-free diet	670

**Table 4:** Meat categories tweeted in connection with the hashtag #foodblogger on Twitter.

Meat Category	Frequency
Poultry *	16,165
Beef	4657
Seafood	6333
Pork	2828
Mutton and Goat	1051

* Poultry: Duck, goose, turkey, and chicken.

**Table 5:** Communities detected in connection with the hashtag #foodblogger on Twitter.

No. Community *	Name of Community	Key Hashtags	Size of Community
1	Healthy lifestyle	Healthylifestyle, vegan, healthyeating, vegetarian, glutenfree, organic, diet	35.92%
2	Home-made food	Tasty, healthy, homemade, dinner, homemadefood, homecooking	32.95%
3	Fast food	Pizza, pasta, burger, delivery, yummy pizzatime, cheatdate, delicious	18.94%
4	Breakfast and brunch	Cake, sweet, chocolate, coffee, baking, cook, desserts, brunch, breakfast	7.56%
5	Food traveling	Travel, travelblogger, travelgram, foodtravel, traveler, travelfoodblog	4.64%

* The community numbers are associated with those shown in Figure 2.

## Data Availability

All data used in this study can be downloaded via Twitter API [78].

## References

[1] Wibowo A., Chen S.-C., Wiangin U., Ma Y., Ruangkanjanases A. (2020). Customer Behavior as an Outcome of Social Media Marketing: The Role of Social Media Marketing Activity and Customer Experience. Sustainability.

[2] Ginsberg K. (2015). Instabranding: Shaping the Personalities of the Top Food Brands on Instagram. Elon J. Undergrad. Res. Commun..

[3] Lee H.-M., Kang J.-W., Namkung Y. (2021). Instagram Users’ Information Acceptance Process for Food-Content. Sustainability.

[4] Walsh M.J., Baker S.A. (2020). Clean Eating and Instagram: Purity, Defilement, and the Idealization of Food. Food Cult. Soc..

[5] Reagan R., Filice S., Santarossa S., Woodrugg J.S. (2020). #ad on Instagram: Investigating the Promotion of Food and Beverage Products. J. Soc. MEDIA Soc..

[6] Roach A. (2002). The Ultimate Guide To The Best Instagram Hashtags For Likes. https://www.oberlo.com/blog/best-instagram-hashtags-for-likes.

[7] Molenaar K. (2022). The Ultimate List of Trending Hashtags on Every Platform. https://influencermarketinghub.com/trending-hashtags/.

[8] Mainolfi G., Marino V., Resciniti R. (2022). Not Just Food: Exploring the Influence of Food Blog Engagement on Intention to Taste and to Visit. Br. Food J..

[9] Hookway N. (2020). Blog Analysis. SAGE Research Methods Foundations.

[10] Lepkowska-White E., Kortright E. (2018). The Business of Blogging: Effective Approaches of Women Food Bloggers. J. Foodserv. Bus. Res..

[11] Pfister D.S. (2014). Networked Media, Networked Rhetorics.

[12] Schmidt J. (2007). Blogging Practices: An Analytical Framework. J. Comput. Commun..

[13] Schneider E.P., McGovern E.E., Lynch C.L., Brown L.S. (2013). Do Food Blogs Serve as a Source of Nutritionally Balanced Recipes? An Analysis of 6 Popular Food Blogs. J. Nutr. Educ. Behav..

[14] Dickinson K., Watson M., Prichard I. (2018). Are Clean Eating Blogs a Source of Healthy Recipes? A Comparative Study of the Nutrient Composition of Foods with and without Clean Eating Claims. Nutrients.

[15] Jia N., Chen J., Wang R. (2022). An Attention-Based Convolutional Neural Network for Recipe Recommendation. Expert. Syst. Appl..

[16] Sthapit E., Piramanayayagam S., Björk P. (2020). Tourists’ Motivations, Emotions, and Memorable Local Food Experiences. J. Gastron. Tour..

[17] Soulis G., Kotsani M., Benetos A. (2019). Let Food and Physical Activity Be Your Medicine. Eur. Geriatr. Med..

[18] Cesiri D. (2020). The Discourse of Food Blogs.

[19] Johnston J., Goodman M.K. (2015). Spectacular Foodscapes. Food Cult. Soc..

[20] Brombin A., Mascarello G., Pinto A., Crovato S., Ricaldi G., Giaretta M., Ravarotto L. (2022). New Ways of Spreading Food Safety Online: The Role of Food Bloggers in Risk Communication. Br. Food J..

[21] Naulin S. (2019). Are food bloggers a new kind of influencer?. Lifestyle Journalism.

[22] Presswood A.L. (2018). Food Blogs, Postfeminism, and the Communication of Expertise: Digital Domestics.

[23] Salvio P.M. (2012). Dishing It Out: Food Blogs and Post-Feminist Domesticity. Gastronomica.

[24] Rodney A., Cappeliez S., Oleschuk M., Johnston J. (2017). The Online Domestic Goddess: An Analysis of Food Blog Femininities. Food Cult. Soc..

[25] Greville-Harris M., Smithson J., Karl A. (2020). What Are People’s Experiences of Orthorexia Nervosa? A Qualitative Study of Online Blogs. Eat. Weight Disord.-Stud. Anorexia Bulim. Obes..

[26] Bissonnette-Maheux V., Provencher V., Lapointe A., Dugrenier M., Dumas A.-A., Pluye P., Straus S., Gagnon M.-P., Desroches S. (2015). Exploring Women’s Beliefs and Perceptions About Healthy Eating Blogs: A Qualitative Study. J. Med. Internet Res..

[27] Boepple L., Thompson J.K. (2014). A Content Analysis of Healthy Living Blogs: Evidence of Content Thematically Consistent with Dysfunctional Eating Attitudes and Behaviors. Int. J. Eat. Disord..

[28] Tiusanen K. (2021). Fulfilling the Self through Food in Wellness Blogs: Governing the Healthy Subject. Eur. J. Cult. Stud..

[29] Véron O. (2016). From Seitan Bourguignon to Tofu Blanquette: Popularizing Veganism in France with Food Blogs. Critical Perspectives on Veganism.

[30] Hart D. (2018). Faux-Meat and Masculinity: The Gendering of Food on Three Vegan Blogs. Can. Food Stud. Rev. Can. Études L’aliment..

[31] Taillon B.J., Mueller S.M., Kowalczyk C.M., Jones D.N. (2020). Understanding the Relationships between Social Media Influencers and Their Followers: The Moderating Role of Closeness. J. Prod. Brand Manag..

[32] Goodman M.K., Jaworska S. (2020). Mapping Digital Foodscapes: Digital Food Influencers and the Grammars of Good Food. Geoforum.

[33] Lofgren J. (2013). Food Blogging and Food-Related Media Convergence. M/C J..

[34] Lynch M., Chamberlain K. (2021). Exploring the food blogosphere. Research Methods in Digital Food Studies.

[35] Germic E.R., Eckert S., Vultee F. (2021). The Impact of Instagram Mommy Blogger Content on the Perceived Self-Efficacy of Mothers. Soc. Media Soc..

[36] Gauthier M., Durocher M. (2018). A Food Blog Created by and for Elders: A Political Gesture Informed by the Normative Injunctions to Eat and Age Well. Interact. Des. Archit..

[37] Diemer S., Brunner M.-L., Schmidt S. (2014). “Like, Pasta, Pizza and Stuff”—New Trends in Online Food Discourse. Cuizine.

[38] Kleindienst D., Pfleger R., Schoch M. The Business Alignment of Social Media Analytics. Proceedings of the ECIS Twenty-Third European Conference on Information Systems.

[39] Pilař L., Kvasničková Stanislavská L., Pitrová J., Krejčí I., Tichá I., Chalupová M. (2019). Twitter Analysis of Global Communication in the Field of Sustainability. Sustainability.

[40] Chen P.-J., Antonelli M. (2020). Conceptual Models of Food Choice: Influential Factors Related to Foods, Individual Differences, and Society. Foods.

[41] Kahneman D. (2003). Maps of Bounded Rationality: Psychology for Behavioral Economics. Am. Econ. Rev..

[42] Decker R., Trusov M. (2010). Estimating Aggregate Consumer Preferences from Online Product Reviews. Int. J. Res. Mark..

[43] Köster E.P. (2003). The Psychology of Food Choice: Some Often Encountered Fallacies. Food Qual. Prefer..

[44] Podsakoff P.M., MacKenzie S.B., Lee J.-Y., Podsakoff N.P. (2003). Common Method Biases in Behavioral Research: A Critical Review of the Literature and Recommended Remedies. J. Appl. Psychol..

[45] Roudsari A., Vedadhir A., Amiri P., Kalantari N., Omidvar N., Eini-Zinab H. (2020). Developing and Validating Food Choice Determinants Questionnaire: An Instrument for Exploring Food Choice Determinants in Iran. Int. J. Prev. Med..

[46] Wilson C. (2013). Questionnaires and Surveys. Credible Checklists and Quality Questionnaires.

[47] Penna A.C.G., Portel C.S., Pagani M.M., Mársico E.T., Silva A.C.O., Esmerino E.A. (2021). Impact of Food Choice and Consumption Profile on the Perception of Food Coloring on Kefir Labels: Insights of the Projective Technique of Product Personality Profiling. Food Res. Int..

[48] Rivaroli S., Calvo-Porral C., Spadoni R. (2022). Using Food Choice Questionnaire to Explain Millennials’ Attitudes towards Craft Beer. Food Qual. Prefer..

[49] Marsola C.d.M., Cunha L.M., Carvalho-Ferreira J.P., da Cunha D.T. (2022). A Dataset of Food Choice Motives among Adults Consumers in Brazil: The Use of Food Choice Questionnaire. Data Br..

[50] Jaeger S.R., Prescott J., Worch T. (2022). Food Neophobia Modulates Importance of Food Choice Motives: Replication, Extension, and Behavioural Validation. Food Qual. Prefer..

[51] Pilař L., Stanislavská L.K., Kvasnička R., Hartman R., Tichá I. (2021). Healthy Food on Instagram Social Network: Vegan, Homemade and Clean Eating. Nutrients.

[52] Pilař L., Kvasničková Stanislavská L., Poláková J., Rojík S., Kvasnička R., Gresham G. (2018). Customer Experience with Organic Food: Global View. Emirates J. Food Agric..

[53] Pilař L., Kvasničková Stanislavská L., Kvasnička R. (2021). Healthy Food on the Twitter Social Network: Vegan, Homemade, and Organic Food. Int. J. Environ. Res. Public Health.

[54] Statista Research Department Social Media-Statistics & Facts. https://www.statista.com/topics/1164/social-networks/#dossierKeyfigures.

[55] Worldometer World Population Projections. https://www.worldometers.info/world-population/world-population-projections/.

[56] Pilař L., Poláková J., Gresham G., Rojík S., Tichá I. Why People Use Hashtags When Visiting Farmers’ Markets. Proceedings of the Agrarian Perspectives XXVI: Competitiveness of European Agriculture and Food Sectors.

[57] Zhang K., Geng Y., Zhao J., Liu J., Li W. (2020). Sentiment Analysis of Social Media via Multimodal Feature Fusion. Symmetry.

[58] Childers C.C., Lemon L.L., Hoy M.G. (2019). #Sponsored #Ad: Agency Perspective on Influencer Marketing Campaigns. J. Curr. Issues Res. Advert..

[59] De Veirman M., Cauberghe V., Hudders L. (2017). Marketing through Instagram Influencers: The Impact of Number of Followers and Product Divergence on Brand Attitude. Int. J. Advert..

[60] Malik A., Berggren W., Al-Busaidi A.S. (2022). Instagram as a Research Tool for Examining Tobacco-Related Content: A Methodological Review. Technol. Soc..

[61] Palazzo M., Vollero A., Vitale P., Siano A. (2021). Urban and Rural Destinations on Instagram: Exploring the Influencers’ Role in #sustainabletourism. Land Use Policy.

[62] Martina C., Ladislav P., Stanislav R. (2021). Organic wine as an Instagram star using a design thinking approach. Transdisciplinary Case Studies on Design for Food and Sustainability.

[63] Vrain E., Wilson C. (2021). Social Networks and Communication Behaviour Underlying Smart Home Adoption in the UK. Environ. Innov. Soc. Transit..

[64] Moreno M.A., Standiford M., Cody P. (2018). Social Media and Adolescent Health. Curr. Pediatr. Rep..

[65] Pilař L., Balcarová T., Rojík S., Tichá I., Poláková J. (2018). Customer Experience with Farmers’ Markets: What Hashtags Can Reveal. Int. Food Agribus. Manag. Rev..

[66] Gascoyne C., Scully M., Wakefield M., Morley B. (2021). Food and Drink Marketing on Social Media and Dietary Intake in Australian Adolescents: Findings from a Cross-Sectional Survey. Appetite.

[67] Molenaar A., Saw W.Y., Brennan L., Reid M., Lim M.S.C., McCaffrey T.A. (2021). Effects of Advertising: A Qualitative Analysis of Young Adults’ Engagement with Social Media About Food. Nutrients.

[68] Pilař L., Kvasničková Stanislavská L., Kvasnička R., Bouda P., Pitrová J. (2021). Framework for Social Media Analysis Based on Hashtag Research. Appl. Sci..

[69] Maynard D., Roberts I., Greenwood M.A., Rout D., Bontcheva K. (2017). A Framework for Real-Time Semantic Social Media Analysis. J. Web Semant..

[70] Arefieva V., Egger R., Yu J. (2021). A Machine Learning Approach to Cluster Destination Image on Instagram. Tour. Manag..

[71] Ashwitha A., Shruthi G., Shruthi H.R., Makarand U., Abhra P.R., Manjunath T.C. (2021). Sarcasm Detection in Natural Language Processing. Mater. Today Proc..

[72] Pilařová L., Kvasničková Stanislavská L., Pilař L., Balcarová T., Pitrová J. (2022). Cultured Meat on the Social Network Twitter: Clean, Future and Sustainable Meats. Foods.

[73] Kvasničková Stanislavská L., Pilař L., Margarisová K., Kvasnička R. (2020). Corporate Social Responsibility and Social Media: Comparison between Developing and Developed Countries. Sustainability.

[74] Chang H.-C., Iyer H. (2012). Trends in Twitter Hashtag Applications: Design Features for Value-Added Dimensions to Future Library Catalogues. Libr. Trends.

[75] Pilař L., Moulis P., Pitrová J., Bouda P., Gresham G., Balcarová T., Rojík S. (2019). Education and Business as a Key Topics at the Instagram Posts in the Area of Gamification. J. Effic. Responsib. Educ. Sci..

[76] Pilař L., Rojík S., Tučková K., Balcarová T., Selby R. Gamification In Education: Social Network Analysis. Proceedings of the 14th International Conference Efficiency And Responsibility In Education, (ERIE).

[77] Twitter Twitter API v2: Early Access. https://developer.twitter.com/en/docs/twitter-api/early-access.

[78] Edward A. An Extensive Guide to Collecting Tweets from Twitter API v2 for Academic Research Using Python 3. https://developer.twitter.com/en/products/twitter-api/academic-research.

[79] Bastian M., Eymann S.H., Jacomy M. Gephi: An Open Source Software for Exploring and Manipulating Networks. Proceedings of the International AAAI Conference on Weblogs and Social Media.

[80] McCurdie T., Sanderson P., Aitken L.M. (2018). Applying Social Network Analysis to the Examination of Interruptions in Healthcare. Appl. Ergon..

[81] Newman M.E.J., Girvan M. (2004). Finding and Evaluating Community Structure in Networks. Phys. Rev. E.

[82] Knoke D., Yang S. (2008). Social Network Analysis.

[83] Blondel V.D., Guillaume J.-L., Lambiotte R., Lefebvre E. (2008). Fast Unfolding of Communities in Large Networks. J. Stat. Mech. Theory Exp..

[84] Muralidhara S., Paul M.J. (2018). #Healthy Selfies: Exploration of Health Topics on Instagram. JMIR Public Health Surveill..

[85] Cavazza N., Graziani A.R., Guidetti M. (2020). Impression Formation via #foodporn: Effects of Posting Gender-Stereotyped Food Pictures on Instagram Profiles. Appetite.

[86] Mejova Y., Haddadi H., Noulas A., Weber I. (2015). #FoodPorn. Proceedings of the 5th International Conference on Digital Health.

[87] Mejova Y., Abbar S., Haddabi H. Fetishizing Food in Digital Age: #foodporn Around the World. Proceedings of the International AAAI Conference on Web and Social Media.

[88] Scherer L., Behrens P., Tukker A. (2019). Opportunity for a Dietary Win-Win-Win in Nutrition, Environment, and Animal Welfare. One Earth.

[89] Bauer J.M., van der Laan L.N., de Bruijn G.-J., Reisch L.A. (2022). Battle of the Primes–The Effect and Interplay of Health and Hedonic Primes on Food Choice. Appetite.

[90] Marty L., Chambaron S., de Lauzon-Guillain B., Nicklaus S. (2022). The Motivational Roots of Sustainable Diets: Analysis of Food Choice Motives Associated to Health, Environmental and Socio-Cultural Aspects of Diet Sustainability in a Sample of French Adults. Clean. Responsible Consum..

[91] De Jans S., Spielvogel I., Naderer B., Hudders L. (2021). Digital Food Marketing to Children: How an Influencer’s Lifestyle Can Stimulate Healthy Food Choices among Children. Appetite.

[92] Santaoja M., Jallinoja P. (2021). Food out of Its Usual Rut. Carnivalesque Online Veganism as Political Consumerism. Geoforum.

[93] Tankovska H. (2017). Daily Social Media Time among Teens and Young Adults Worldwide. https://www.statista.com/statistics/800821/average-daily-time-spent-social-media-teens-young-adults/.

[94] North M., Kothe E., Klas A., Ling M. (2021). How to Define “Vegan”: An Exploratory Study of Definition Preferences among Omnivores, Vegetarians, and Vegans. Food Qual. Prefer..

[95] Stremmel G., Elshiewy O., Boztug Y., Carneiro-Otto F. (2022). Vegan Labeling for What Is Already Vegan: Product Perceptions and Consumption Intentions. Appetite.

[96] Saboia I., Pisco Almeida A.M., Sousa P., Pernencar C. (2018). I Am with You: A Netnographic Analysis of the Instagram Opinion Leaders on Eating Behavior Change. Procedia Comput. Sci..

[97] Van Bavel J.J., Rathje S., Harris E., Robertson C., Sternisko A. (2021). How Social Media Shapes Polarization. Trends Cogn. Sci..

[98] Bruns A. (2019). After the ‘APIcalypse’: Social Media Platforms and Their Fight against Critical Scholarly Research. Inf. Commun. Soc..

